# Electron Paramagnetic Resonance Study of Thermally Treated Bismuth Subgallate

**DOI:** 10.1155/2014/547032

**Published:** 2014-11-26

**Authors:** Paweł Ramos, Barbara Pilawa

**Affiliations:** School of Pharmacy with the Division of Laboratory Medicine in Sosnowiec, Department of Biophysics, Medical University of Silesia in Katowice, Jedności 8, 41-200 Sosnowiec, Poland

## Abstract

Complex of bismuth, an anti-inflammatory drug, was studied by EPR spectroscopy. The aim of this study was to determine concentrations and properties of free radicals formed during thermal sterilization of bismuth subgallate according to pharmacopoeia norms to optimize its sterilization process. Different temperatures (160°C, 170°C, and 180°C) and times (120 minutes, 60 minutes, and 30 minutes) of sterilization were used. Interactions of bismuth subgallate with DPPH, the model free radical reference, were checked. *g*-Factors, amplitudes (*A*), integral intensities (*I*), and linewidths (Δ*B*_pp_) were obtained. Integral intensities were obtained by double integration of the first-derivative EPR lines. The influence of microwave power in the range of 2.2–70 mW on shape and parameters of the EPR spectra was examined. Thermal sterilization produced free radicals in bismuth subgallate in all tested cases. Strong interactions with free radicals were pointed out for all the analysed samples containing bismuth independent of sterilization conditions. Optimal conditions of thermal sterilization for bismuth subgallate with the lowest free radical formation are temperature 170°C and time of heating 60 minutes. Strong dipolar interactions exist in thermally sterilized bismuth subgallate. EPR spectroscopy is a useful method of examination of thermal sterilization conditions.

## 1. Introduction

Electron paramagnetic resonance (EPR) spectroscopy is the useful method in pharmacy [1–5] and medicine [6, 7]. Studies of free radicals in sterilized drugs [1–5], cells [8], and tissues [7] have been carried out before. EPR studies were done for melanin biopolymers and their complexes with metal ions [9–11] and drugs [12]. Metal ions modify formation of melanin complex with drugs [13]. Cooper (II) decreases and zinc (II) increases the amounts of o-semiquinone free radicals in melanins, respectively [10]. In this work electron paramagnetic resonance was used to study the heated exemplary drug containing bismuth. Both free radical contents and interactions of this drug with free radicals were examined.

Complex of bismuth reveals antibacterial properties, and this metal is the component in modern anti-inflammatory drugs [14, 15]. Drugs should not introduce microorganisms into cells, and they are sterilized during production [16, 17]. The method of sterilization is fitted to chemical structure of the drug. Thermally stabile substances may be sterilized at high temperatures, which kill bacteria, viruses, fungi, and the other microorganisms [16, 17]. The effect of high temperature on sterilized drug should not produce high amounts of free radicals in the sample. Free radicals containing unpaired electrons destroy biological structures in living organism [18]. We proposed EPR measurements for drugs as an additional test. Searches were optimal conditions of temperature and heating times of solid samples, which give the lowest concentration of free radicals. We expected the lowest EPR signals for the optimally sterilized drug. In this work the efficacy of thermal sterilization for bismuth subgallate was spectroscopically checked. The aim of this study was to determine free radical concentrations and properties of free radicals formed during thermal sterilization of bismuth subgallate at different temperatures and times. The information about free radicals in heated bismuth subgallate is helpful to optimize its sterilization process.

## 2. Experimental

### 2.1. Characterization and Preparation of the Samples

**Figure 1:**
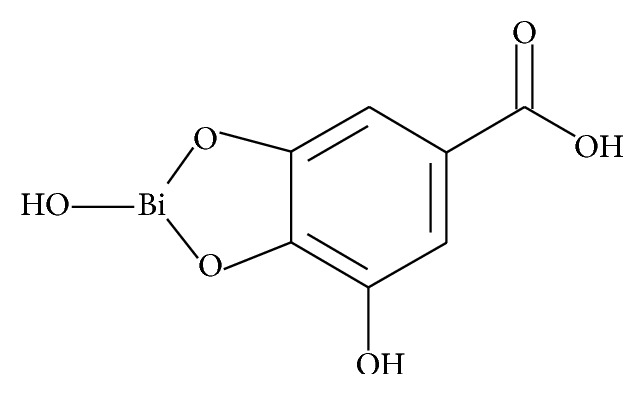
Chemical structure of bismuth subgallate [19].

Thermally sterilized bismuth subgallate was examined in this work. The tested drug containing bismuth used in medicine as a powder on the skin is used as a disinfectant, desiccant, astringent, and anti-inflammatory [14, 15]. Bismuth subgallate can be used orally as an antidiarrheal agent and screening mucosa of stomach and duodenum [14, 15]. Bismuth subgallate is used as an ingredient in ointments and powders in skin diseases such as inflammation of the skin, oozing wounds, and ulcers [14, 15]. Chemical structure of bismuth subgallate is shown in Figure 1 [19].

Thermal treatment was chosen as a sterilization method. Bismuth subgallate was sterilized in hot air according to the pharmacopoeia norms [17] at temperatures 160°C, 170°C, and 180°C. Times of heating of the drug sample were 30 minutes, 60 minutes, and 120 minutes, respectively. The professional apparatus for thermal sterilization in hot air produced by Memmert Firm (Germany) was used in the experiment. The samples in the solid state were located in this apparatus and heated at temperatures and times from the norms. The microorganisms are absent in the samples sterilized according to the norms [17]. We performed EPR examination to choose from these conditions the parameters (temperature and time of heating), which produce the lowest concentration in the heated samples.

To examine free radicals the powdered heated bismuth subgallate samples were located in thin walled glass tubes with the external diameter of 3 mm, and the EPR spectra were measured. Free radicals in the thermally sterilized drug were tested 20 minutes after sterilization.

The interactions of the heated bismuth subgallate with free radicals were examined by the use of DPPH (2,2-diphenyl-1-picrylhydrazyl) as the model free radical reference. DPPH solutions in ethyl alcohol with concentration 0.1 mM were prepared. The ethyl alcohol solutions of DPPH at the presence of the individual bismuth samples were done.

### 2.2. EPR Measurements

**Figure 2:**
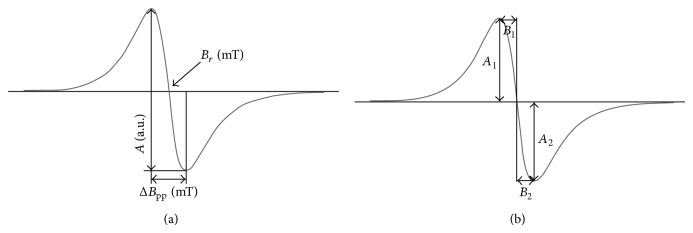
The analysed parameters of the first-derivative EPR spectra (a): *A*—amplitude, Δ*B*_pp_—line width, and *B*_*r*_—resonance magnetic induction and the tested spectral shape parameters (b): *A*_1_, *A*_2_, *B*_1_, and *B*_2_, (b).

The electron paramagnetic resonance measurements of bismuth subgallate were done at room temperature. An X-band (9.3 GHz) EPR spectrometer with magnetic modulation of 100 kHz produced by Radiopan Firm (Poznań, Poland) was used. The numerical acquisition of the EPR spectra was performed by the Rapid Scan Unit of Jagmar Firm (Kraków, Poland). The klystron of the spectrometer produced total microwave power of 70 mW. The microwave power was changed by applying different attenuations (up to 15 dB). First-derivative EPR spectra were recorded with the microwave powers in the range of 2.2–70 mW. Line shape and parameters of the EPR spectra were analysed as shown in Figure 2.

*g*-Factors, amplitudes (*A*), integral intensities (*I*), and line widths (Δ*B*_pp_) were obtained. Integral intensity was obtained by double integration of the first-derivative EPR spectra. The influence of microwave power on line shape and parameters of the EPR spectra of all the samples were tested. *g*-Factor characterized localization of unpaired electrons in the samples was calculated from the paramagnetic resonance condition as [20]

(1) $$\begin{array}{c} g=\frac{h\nu }{\mu_{B}B_{r}}, \end{array}$$

where *h*—Planck constant, *ν*—microwave frequency, *μ*_*B*_—Bohr magneton, and *B*_*r*_—induction of resonance magnetic field.

*g*-Factors were near 2 for all the tested samples.

Free radical concentrations (*N*) in bismuth subgallate sterilized at different conditions were determined. Ultramarine and a ruby crystal were the references. The spectroscopic programs of Jagmar Firm and LabView (National Instruments) were used. Free radical concentrations (*N*) in the tested samples were determined according to the formula [20]

(2) $$\begin{array}{c} N=N_{u}\left[\frac{\left(W_{u}A_{u}\right)}{I_{u}}\right]\cdot \left[\frac{I}{\left(WAm\right)}\right], \end{array}$$

where *N*_*u*_—the number of paramagnetic center in the ultramarine reference; *W*, *W*_*u*_—the receiver gains for sample and the ultramarine; *A*, *A*_*u*_—the amplitudes of ruby signal for the sample and the ultramarine; *I*, *I*_*u*_—the integral intensities for the sample and ultramarine; *m*—the mass of the sample.

The integral intensities (*I*) of the EPR lines of the analysed drug were compared to those of ultramarine, the reference. A ruby crystal (Al_2_O_3_ : Cr^3+^) was used as the second reference. It was permanently placed in a resonance cavity during the measurements of the EPR lines for both bismuth subgallate and ultramarine.

The effect of microwave power on amplitudes (*A*) and line widths (Δ*B*_pp_) of EPR spectra was determined. The correlations between amplitudes (*A*), line widths (Δ*B*_pp_), and microwave power let as conclude about homogeneous or inhomogeneous line broadening. For homogeneous distribution of free radicals in the samples the amplitude (*A*) increases with increasing microwave power (*M*) and for the higher microwave powers it decreases [20]. Line width (Δ*B*_pp_) of the homogeneously broadened EPR lines increases with increasing of microwave power [20]. For inhomogeneously broadened lines the amplitude (*A*) increases with increasing of microwave power (*M*) and for the higher microwave powers its value is constant [20]. Line width (Δ*B*_pp_) of the inhomogeneously broadened EPR lines does not depend on microwave power [20]. The correlations between amplitudes and microwave power characterize spin-lattice relaxation processes. Amplitudes of EPR lines of the samples with the faster spin-lattice relaxation processes decrease at the higher microwave powers [20].

EPR lines of the model free radicals of DPPH and DPPH interacting with the tested bismuth samples were obtained. Next EPR lines for DPPH solution in ethyl alcohol at the presence of the tested samples were detected. The quenching of the model free radicals by the tested dermatological sample causes decreasing of the amplitude (*A*) and integral intensity (*I*) of EPR line of DPPH.

## 3. Results and Discussion

**Figure 3:**
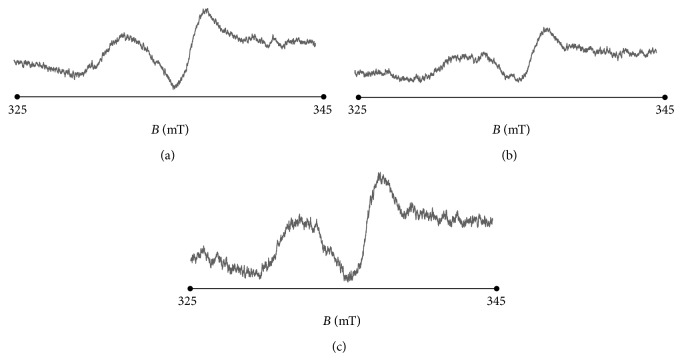
EPR spectra of bismuth subgallate thermally sterilized at 160°C (120 minutes), 170°C (60 minutes), and 180°C (30 minutes). *B*—magnetic induction. The spectra were measured with attenuation of 15 dB at room temperature.

**Figure 4:**
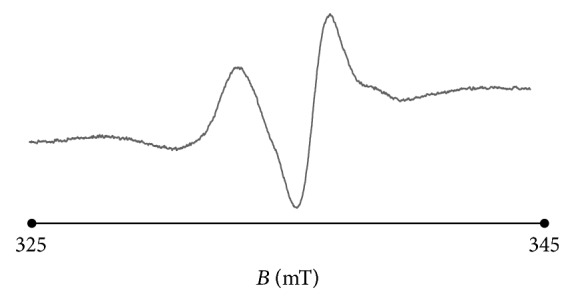
EPR spectra of bismuth subgallate thermally sterilized at 160°C (120 minutes). *B*—magnetic induction. The spectra were measured with attenuation of 15 dB at liquid nitrogen temperature.

All the tested, thermally treated bismuth subgallate samples were paramagnetic exhibiting EPR bands with complex shape (Figure 3). In Figure 3 the spectra of bismuth subgallate heated during different times at the tested temperatures are shown. The used parameters of thermal treatment are determined by pharmaceutical norms [17]. EPR spectra of heated bismuth subgallate obtained at liquid nitrogen temperature are presented in Figure 4.

The exemplary EPR spectrum of bismuth subgallate heated at temperature 160°C during 120 minutes obtained at liquid nitrogen temperature is presented in Figure 4. EPR spectra of bismuth subgallate revealed complex shape both at room (Figure 3) and liquid nitrogen (Figure 4) temperatures. Thermally formed free radicals existed in the tested samples. It seems possible that additional signals of bismuth will appear in the EPR spectra. It is interesting to examine the bismuth signals in the future work. In this study we concentrated on free radicals in the pharmaceutical samples at the presence of bismuth.

**Figure 5:**
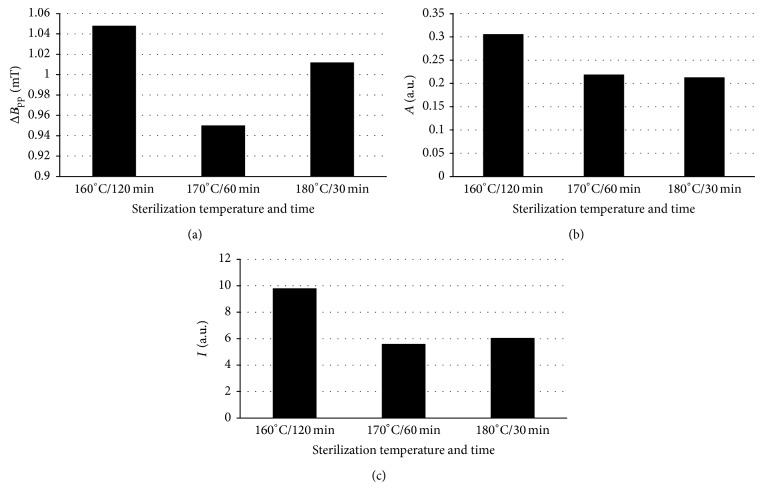
The parameters of the EPR spectra bismuth subgallate thermally sterilized at 160°C (120 minutes), 170°C (60 minutes), and 180°C (30 minutes): (a) line widths (Δ*B*_pp_), (b) amplitudes (*A*), and (c) integral intensities (*I*). The comparison was done for the spectra measured with attenuation of 15 dB.

EPR results for bismuth subgallate examined at room temperature are presented below. Parameters of the EPR spectra of bismuth subgallate depended on temperature and time of thermal sterilization. The parameters of the EPR lines of bismuth subgallate thermally sterilized at temperatures 160°C (120 minutes), 170°C (60 minutes), and 180°C (30 minutes), line widths (Δ*B*_pp_), amplitudes (*A*), and integral intensities (*I*) are compared in Figures 5(a)–5(c). The high line widths (Δ*B*_pp_) were in the range of 0.95–1.048 mT (Figure 5(a)). This line broadening is caused by strong dipolar interactions of unpaired electrons in the bismuth samples [20]. The strong dipolar interactions are characteristic for unpaired electrons nearly located. The lowest line width (Δ*B*_pp_) was obtained for bismuth subgallate sterilized at 170°C during 60 minutes (Figure 5(a)), so the relatively highest distances between unpaired electrons exist in this sample. The relatively broader EPR lines were detected for bismuth subgallate sterilized at 160°C during 120 minutes and 180°C during 30 minutes (Figure 5(a)), and it indicates the relatively lowest distances between unpaired electrons in these samples compared to the sample heated at 170°C during 60 minutes (Figure 5(a)). The lowest and the highest amplitudes (*A*) were observed for the EPR lines of bismuth subgallate sterilized at 180°C and 160°C, respectively (Figure 5(b)). The lowest and the highest integral intensities (*I*) were observed for the EPR lines of bismuth subgallate sterilized at 170°C and 160°C, respectively (Figure 5(c)). These parameters implicated different values of free radical concentrations in the tested samples thermally treated at 160°C, 170°C, and 180°C.

**Figure 6:**
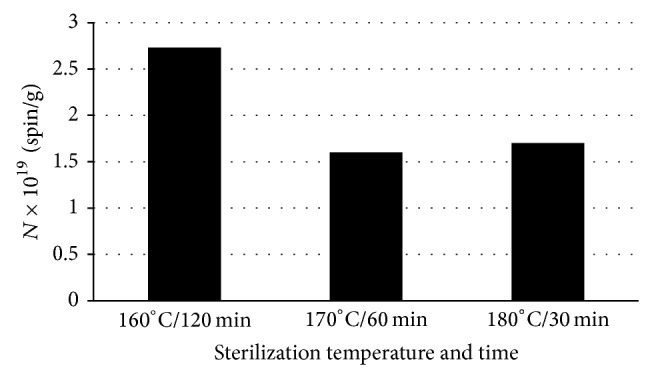
Free radical concentrations (*N*) in bismuth subgallate thermally sterilized at 160°C (120 minutes), 170°C (60 minutes), and 180°C (30 minutes).

Free radical concentrations in bismuth subgallate thermally sterilized at temperatures 160°C (120 minutes), 170°C (60 minutes), and 180°C (30 minutes) are compared in Figure 6. The lowest free radical concentration appeared in bismuth subgallate sterilized at 170°C during 60 minutes, while the highest free radical concentration existed in bismuth subgallate sterilized at 160°C during 120 minutes. Among the studied conditions, the most optimal one for sterilizing bismuth subgallate is 170°C, based on EPR measurements.

**Figure 7:**
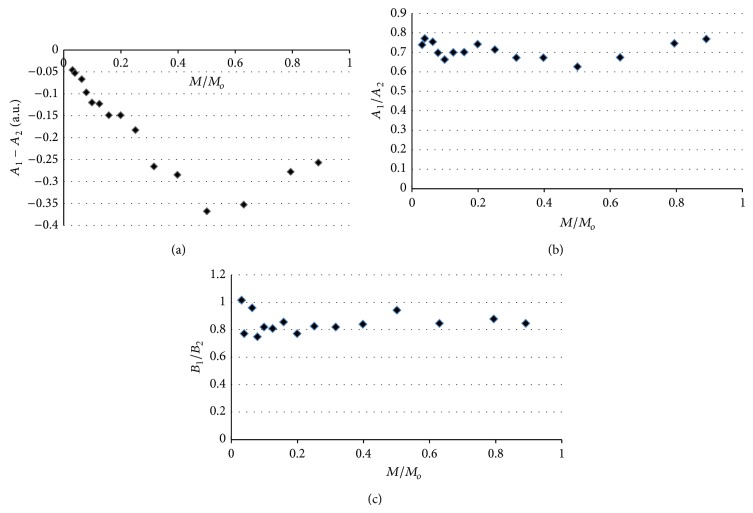
The effect of microwave power on the line shape parameters: (a) *A*_1_ − *A*_2_, (b) *A*_1_/*A*_2_, and (c) *B*_1_/*B*_2_, for the EPR spectra of bismuth subgallate thermally sterilized at 160°C (120 minutes). *M*—microwave power used during the measurement of the EPR spectra, *M*_*o*_—total microwave power produced by klystron (70 mW).

**Figure 8:**
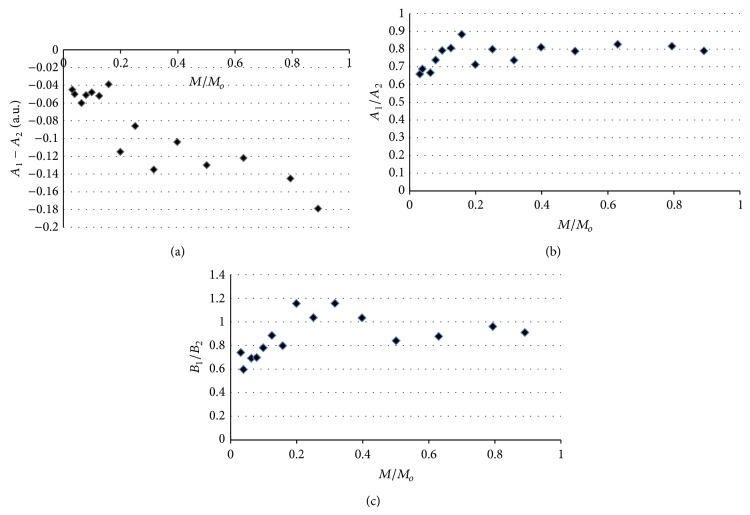
The effect of microwave power on the line shape parameters: (a) *A*_1_ − *A*_2_, (b) *A*_1_/*A*_2_, and (c) *B*_1_/*B*_2_, for the EPR spectra of bismuth subgallate thermally sterilized at 170°C (60 minutes). *M*—microwave power used during the measurement of the EPR spectra, *M*_*o*_—total microwave power produced by klystron (70 mW).

**Figure 9:**
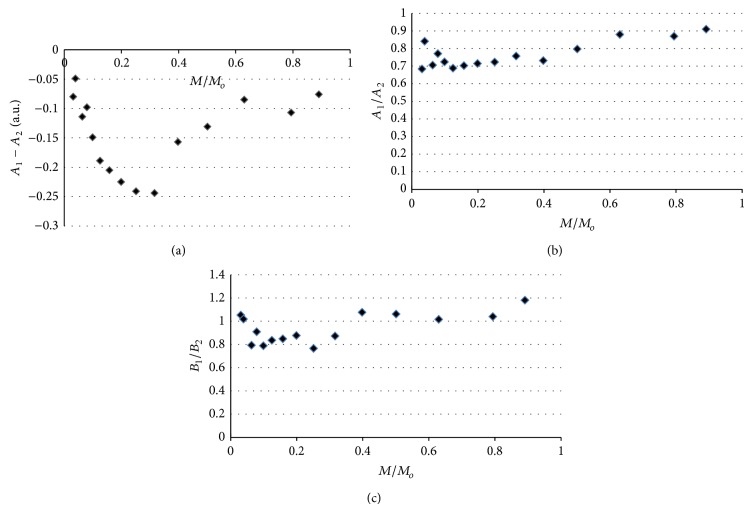
The effect of microwave power on the line shape parameters: (a) *A*_1_ − *A*_2_, (b) *A*_1_/*A*_2_, and (c) *B*_1_/*B*_2_, for the EPR spectra of bismuth subgallate thermally sterilized at 180°C (30 minutes). *M*—microwave power used during the measurement of the EPR spectra, *M*_*o*_—total microwave power produced by klystron (70 mW).

The shape of the EPR spectra of thermally sterilized bismuth subgallate changed with increasing of microwave power used during the detection of lines. The effect of microwave power on the line shape parameters *A*_1_ − *A*_2_, *A*_1_/*A*_2_, and *B*_1_/*B*_2_, for the EPR spectra of bismuth subgallate thermally sterilized at 160°C (120 minutes), 170°C (60 minutes), and 180°C (30 minutes), is presented in Figures 7(a)–7(c), 8(a)–8(c), and 9(a)–9(c), respectively. The asymmetry parameters of the EPR spectra are shown (Figures 7–9). The changes of the asymmetry of these spectra resulted from existence of several groups of free radicals in the heated drug. The individual lines of different types of free radicals changed with increasing of microwave power in the manner characteristic for them, so the shapes of the resultant spectra were not stabile. Formation of several types of free radicals in bismuth subgallate was expected, because of rupturing of different chemical bonds at high temperatures.

**Figure 10:**
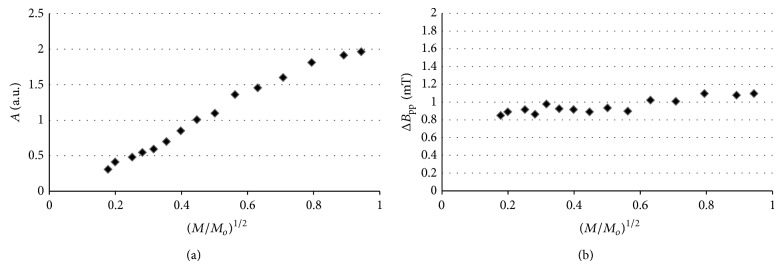
The effect of microwave power on (a) amplitude (*A*) and (b) line width (Δ*B*_pp_), for the EPR spectra of bismuth subgallate thermally sterilized at 160°C (120 minutes). *M*—microwave power used during the measurement of the EPR spectra, *M*_*o*_—total microwave power produced by klystron (70 mW).

**Figure 11:**
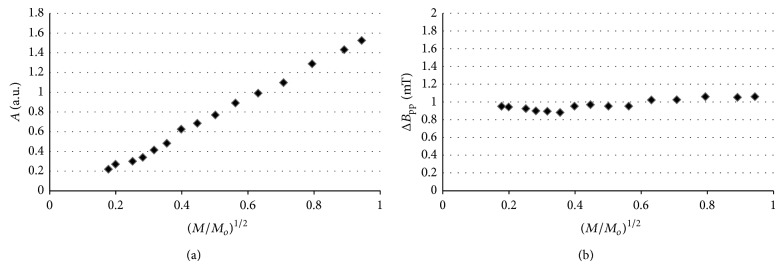
The effect of microwave power on (a) amplitude (*A*) and (b) line width (Δ*B*_pp_), for the EPR spectra of bismuth subgallate thermally sterilized at 170°C (60 minutes). *M*—microwave power used during the measurement of the EPR spectra, *M*_*o*_—total microwave power produced by klystron (70 mW).

**Figure 12:**
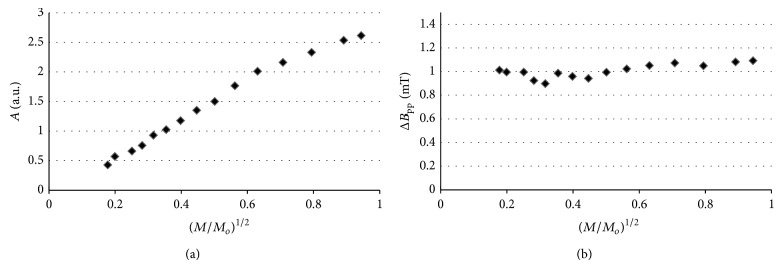
The effect of microwave power on (a) amplitude (*A*) and (b) line width (Δ*B*_pp_), for the EPR spectra of bismuth subgallate thermally sterilized at 180°C (30 minutes). *M*—microwave power used during the measurement of the EPR spectra, *M*_*o*_—total microwave power produced by klystron (70 mW).

Amplitudes (*A*) and line widths (Δ*B*_pp_) of the EPR spectra of thermally sterilized bismuth subgallate changed with microwave power. The effect of microwave power on amplitudes (*A*) and line widths (Δ*B*_pp_), for the EPR spectra of the tested drug heated at 160°C (120 minutes), 170°C (60 minutes), and 180°C (30 minutes), is shown in Figures 10(a), 10(b), 11(a), 11(b), 12(a), and 12(b), respectively. Line widths (Δ*B*_pp_) for all the measured EPR lines increased with increasing of microwave power (Figures 10(a)–12(a)). Such effect is characteristic for EPR lines homogeneously broadened [20]. Amplitudes (*A*) increased with increasing of microwave power for bismuth subgallate sterilized at all the tested conditions, and the microwave saturation effect was not observed (Figures 10(b)–12(b)). The correlation between amplitudes (*A*) and microwave power are characteristic for fast spin-lattice relaxation processes in the samples [20]. Homogeneously broadened EPR lines [1–5] for sterilized drugs and fast spin-lattice relaxation processes [2–4] were observed by as earlier.

**Figure 13:**
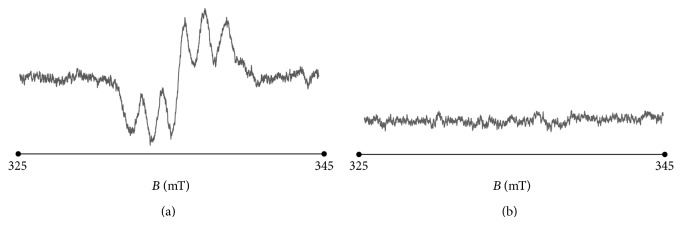
The EPR spectrum of (a) ethyl alcohol solution of DPPH and (b) the quenched line after addition of bismuth subgallate sterilized at temperature 180°C (30 minutes) to this solution.

The thermally sterilized bismuth subgallate samples strongly interact with free radicals. The susceptibility of the sample to interactions with free radicals are usually tested with the model paramagnetic reference [7, 20]. DPPH with free radicals with unpaired electrons localized on nitrogen atom is used as the model of the source of free radicals. EPR lines of nitrogen free radicals of DPPH in ethyl alcohol solution decreased after addition of the tested sample, bismuth subgallate. The interactions of the sample with free radicals of DPPH increase with increasing of this decrease of amplitude of the EPR line of DPPH. The interactions quenching the EPR signals of DPPH are the result of formation of pair of electrons by one electron of DPPH and one electron of the tested sample. Quenching of free radicals by pharmaceutical samples is the positive effect, because free radicals in living organism are responsible for negative effects destroying tissues [7, 18]. The exemplary EPR spectrum of ethyl alcohol solution of DPPH and its quenching after addition of bismuth subgallate sterilized at temperature 180°C during 30 minutes to this solution is presented in Figures 13(a) and 13(b), respectively. Such quenching of free radicals of DPPH was observed for all the analysed bismuth subgallate samples. Quenching of free radicals will be the positive property of bismuth subgallate during its application in living organism.

The performed studies confirmed usefulness of an X-band (9.3 GHz) electron paramagnetic resonance spectroscopy to certification of harmless properties of pharmaceutical samples containing bismuth.

## 4. Conclusions

The performed electron paramagnetic resonance studies of thermally sterilized bismuth subgallate pointed out the following.

- Free radicals are formed in bismuth subgallate sterilized at 160°C (120 minutes), 170°C (60 minutes), and 180°C (30 minutes).
- Optimal conditions of thermal sterilization for bismuth subgallate with the lowest free radical formation are temperature 170°C and time of heating 60 minutes.
- Strong dipolar interactions and fast spin-lattice relaxation processes exist in thermally sterilized bismuth subgallate.
- EPR spectroscopy can be a useful method for certification of harmless properties of samples containing bismuth complexes.
